# Evaluation of the Cytotoxicity of Structurally Correlated *p*-Menthane Derivatives

**DOI:** 10.3390/molecules200713264

**Published:** 2015-07-21

**Authors:** Luciana Nalone Andrade, Tamires Cardoso Lima, Ricardo Guimarães Amaral, Cláudia do Ó Pessoa, Manoel Odorico de Moraes Filho, Bruno Marques Soares, Lázaro Gomes do Nascimento, Adriana Andrade Carvalho, Damião Pergentino de Sousa

**Affiliations:** 1 Departamento de Fisiologia, Universidade Federal de Sergipe, CEP 49100-000, São Cristóvão-SE, Brazil; E-Mails: lulisynalone@yahoo.com.br (L.N.A.); tam_tamires@yahoo.com.br (T.C.L.); ricardoamaral23@hotmail.com (R.G.A.); 2 Departamento de Fisiologia e Farmacologia, Universidade Federal do Ceará, CEP 60430-270, Fortaleza-SE, Brazil; E-Mails: caudoo05@hotmail.com (G.P.); odorico@ufc.br (M.O.M.F.); brunomsoares@gmail.com (B.M.S.); 3 Universidade Federal da Paraíba, CP 5009, CEP 58051-970, João Pessoa-PB, Brazil; E-Mail: lazarofarm2@gmail.com; 4 Universidade Federal de Sergipe, CEP 58051-970, Lagarto-SE, Brazil; E-Mail: a.acarvalho@yahoo.com.br

**Keywords:** cytotoxic activity, cytotoxicity, essential oils, monoterpenes, *p*-menthane, natural products, anticancer, antitumoral, perillyl alcohol

## Abstract

Compounds isolated from essential oils play an important role in the prevention and treatment of cancer. Monoterpenes are natural products, and the principal constituents of many essential oils. The aim of this study was to investigate the cytotoxic potential of *p*-menthane derivatives. Additionally, analogues of perillyl alcohol, a monoterpene with known anticancer activity, were evaluated to identify the molecular characteristics which contribute to their cytotoxicity, which was tested against OVCAR-8, HCT-116, and SF-295 human tumor cell lines, using the MTT assay. The results of this study showed that (−)-perillaldehyde 8,9-epoxide exhibited the highest percentage inhibition of cell proliferation (GI = 96.32%–99.89%). Perillyl alcohol exhibited high cytotoxic activity (90.92%–95.82%), while (+)-limonene 1,2-epoxide (GI = 58.48%–93.10%), (−)-perillaldehyde (GI = 59.28%–83.03%), and (−)-8-hydroxycarvotanacetone (GI = 61.59%–94.01%) showed intermediate activity. All of the compounds tested were less cytotoxic than perillyl alcohol, except (−)-perillaldehyde 8,9-epoxide (IC_50_ = 1.75–1.03 µL/mg). In general, replacement of C-C double bonds by epoxide groups in addition to the aldehyde group increases cytotoxicity. Furthermore, stereochemistry seems to play an important role in cytotoxicity. We have demonstrated the cytotoxic influence of chemical substituents on the *p*-menthane structure, and analogues of perillyl alcohol.

## 1. Introduction

Cancer is a disease in which certain cells in the body grow in an uncontrolled way. It is one of the world’s most serious illnesses, and together with heart attacks, it kills more people than any other disease. Its hallmarks constitute an organizing principle for rationalizing the complexities of neoplastic diseases. They include sustained proliferative signaling, evading growth suppressors, resisting cell death, enabling replicative immortality, inducing angiogenesis, and activating invasion and metastasis [1].

Nature is an important source of new candidates for therapeutic compounds; a large chemical diversity is found in several species of plants, animals, and microorganisms [2]. Prakash and collaborators [3] have reported that natural products, since ancient times, have been used for health purposes by all cultures, as well as being a source of medicines. It has been estimated that about 80%–85% of the global population relies on traditional medicines for their primary health care needs, and it is assumed that a major part of traditional therapy involves the use of plant extracts or their active principles [4,5,6]. Although many recent investigations and advancements in the treatment and control of cancer progression have been carried out, significant work and room for improvement remain. The main disadvantage of synthetic drugs is their associated side effects. However, natural therapies, that use plants or plant-derived natural products are being found beneficial to combat cancer [3]. The search for antitumor agents from plant sources started in the 1950s with the discovery and development of the vinca alkaloids (vinblastin and vincristine), and the isolation of other cytotoxic compounds [7].

Antitumor activity has been reported for essential oils against several tumor cell lines [8,9,10,11]. Essential oils have been demonstrated to have antitumor activity in a variety of cell lines, and this is attributed to their chemical constituents, including monoterpenes such as myrcene [12], citronellol [13], terpinen-4-ol [14], and limonene [15], which are found respectively in *Vepris macrophylla* and *Myristica fragrans* [16].

Some antitumor essential oils have a high percentage of monoterpenes in their chemical compositions. These substances often contribute to the pharmacological activity of these essential oils [16]. Monoterpenes are found in the essential oils of many plants including fruits, vegetables, and herbs. They have been shown to have a large number of diverse cellular and molecular effects both *in vitro* and *in vivo*, in addition to preventing the process of carcinogenesis at both the initiation and the promotion/progression stages [16,17]. They are effective in treating early and advanced cancers. Because of results like these, about 74% of drugs in the anticancer area are either natural products or derived from natural products [18].

Compounds such as thymol, perillic acid, and perillyl alcohol, among others, have been shown to prevent mammary, liver, lung, and other cancers [16,19]. Perillyl alcohol, a naturally occurring monoterpene found in lavender, cherries, and mint, has been suggested as an effective agent against a variety of tumors [20,21]. Perillyl alcohol has shown antitumor activity against pancreatic carcinomas [22,23], anti-metastatic activity in a chorioallantoic membrane model [24], and it inhibits the proliferation of human adenocarcinoma (A549), squamous cell carcinoma (H520) [21], and *in vitro* cultured A549 and BroTo cells [25]. Among the monoterpenes perillyl alcohol is thus one of the most promising anticancer agents. As a chemotherapeutic agent it has advanced to phase II clinical trials in cancer patients, and the preliminary results indicate that it is well tolerated [26,27].

According to Sobral and collaborators [16], many studies evaluate the cytotoxic activity of essential oils, and the isolation of their main constituents, but do not correlate their chemical structures with biological activity. Therefore considering the anticancer bioactivity of perillyl alcohol, this study aims to evaluate the cytotoxic activity of 18 *p*-menthane derivatives structurally correlated with perillyl alcohol, and to investigate their structure-activity relationships against human tumor cell lines.

## 2. Results and Discussion

### 2.1. Antiproliferative Effect of Compounds

**Figure 1:**
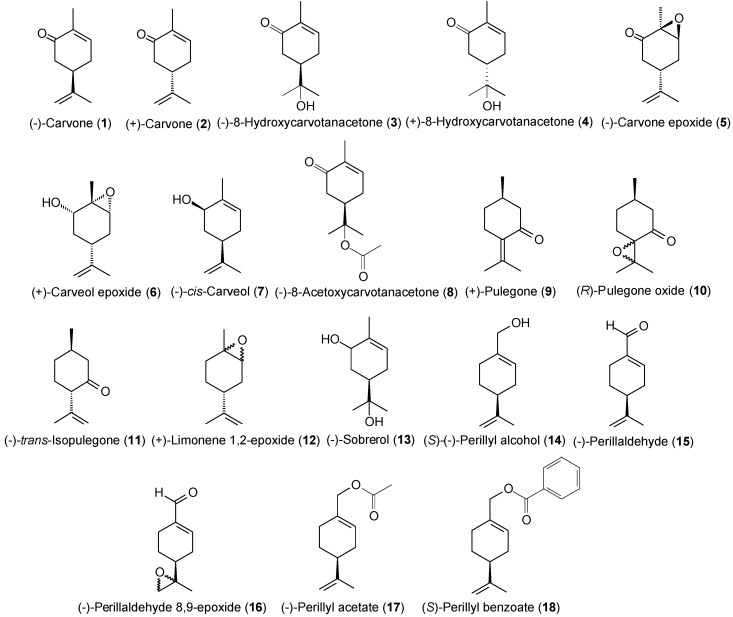
Structures of evaluated compounds.

Based on the excellent cytotoxic activity of perillyl alcohol and several monoterpenes found in essential oils, we chose 18 structurally correlated compounds (Figure 1) to evaluate their cytotoxic activity against tumor cell lines and establish the corresponding SAR.

The cytotoxic activity of the eighteen monoterpenes was evaluated against several human tumor cell lines: HCT-116 (colon), OVCAR-8 (ovarian), and SF-295 (brain). In the screening program for the discovery and development of potential anticancer of compounds, the criteria of the American National Cancer Institute were adopted for the selection of cells [2,28,29]. The results were assessed by comparing the cell growth inhibition percentage (GI%) values, expressed in percentage. (GI%) values are presented as the mean ± SD of three replicates measured by MTT assay after 72 h of incubation, as summarized in Table 1. The results of the cytotoxicity assays revealed that all of the test compounds exhibit cytotoxic activity against these tumor cell lines, as shown in Table 1. The results were evaluated using an intensity scale for each tested cell line as follows: samples with weak cytotoxic activity (GI < 50%), intermediate activity (GI 51%–75%), and high activity (GI > 75%) [30]. The compounds **1**, **2**, **4**, **5**, **6**, **7**, **8**, **9**, **10**, **11**, **12**, **13**, **17** and **18** demonstrated GI < 50% for the three cell lines studied, being classified as compounds with weak cytotoxic activity.

Compound **16** exhibited high cytotoxicity when compared with the other monoterpenes, with a GI value ranging from 96.32%–99.89%, inducing almost 100% mortality in the cells, at a concentration of 25 µg/mL. A comparison of perillyl alcohol and its analogues showed that all tested analogues (Figure 1) were found to have less potent cytotoxic activities than perillyl alcohol itself (Table 1), except for **16**. Compound **16**, an aldehyde monoterpene, was significantly the most cytotoxic compound (GI = 96.32%–99.89%), followed by perillyl alcohol (**14**) (GI = 90.92%–95.82%), (−)-8-hydroxy-carvotanacetone (**3**) (GI = 61.59%–94.01%). (+)-limonene 1,2-epoxide (**12**) (GI = 58.48%–93.10%) and (−)-perillaldehyde (**15**) (GI = 59.28%–83.03%), which exhibited cytotoxic activity ranging between high and intermediate depending on the cell line observed, as described in Table 1.

In *in vitro* cell culture systems, cytotoxic compounds interfere with cellular attachment, resulting in significant alteration in morphology, adversely affecting cell growth rate, or causing cell death [31]. Therefore, the cell proliferation assays were performed with structurally correlated compounds. This experimental model is a well-characterized colorimetric assay that is based on the enzymatic reduction of the tetrazolium salt MTT in living, metabolically active cells, but not in dead cells. It has been widely used to determine the cytostatic/cytotoxic potential of medicinal agents in screening programs [28,29,32]. Among the five structurally related compounds with cytotoxic activity ranging from intermediate to high, the compounds **12** [33], **14** [21], and **15** [25] exhibit cytotoxic activity and are already described in the literature. Therefore, we determined the median inhibitory concentration able to induce 50% of maximal effect (IC_50_) of the compounds **3** and **16**, which have no cytotoxic activity described in the literature. Since compound **16** was more potent, it was subjected to additional evaluation of its cytotoxic activity against HL-60, with the intention of assessing the cell death process. The HL-60 cell is more sensitive to chemotherapeutic agents, with well-defined methods for checking cellular death processes [34,35,36].

**Table 1:** Cell growth inhibition percentage of compounds tested at the concentration of 25 μg/mL against tumor cell lines.

Compounds	Cells					
	HCT-116	SD	OVCAR-8	SD	SF-295	SD
	IG%		IG%		IG%	
(−)-Carvone (**1**)	**11.94**	±2.54	**2.28**	±1.38	**12.28**	±1.13
(+)-Carvone (**2**)	**46.15**	±2.46	**48.07**	±1.20	**34.39**	±3.47
(−)-8-Hydroxycarvotanacetone (**3**)	**75.2**	±2.62	**94.01**	±1.38	**61.59**	±3.10
(+)-8-Hydroxycarvotanacetone (**4**)	**4.76**	±1.85	**3.12**	±2.96	**16.36**	±1.07%
(−)-Carvone epoxide (**5**)	**29.24**	±1.00	**8.21**	±0.49	**10.93**	±0.06
(+)-Carveol epoxide (**6**)	**12.43**	±4.31	**4.58**	±8.58	**35.35**	±2.44
(−)- *cis*-Carveol (**7**)	**9**	±2.38	**3.61**	±9.96	**21.16**	±1.19
(−)-8-Acetoxycarvotanacetone (**8**)	**10.36**	±9.38	**1.62**	±1.58	**30.47**	±3.51
(+)-Pulegone (**9**)	**10.25**	±5.85	**14.41**	±8.08	**27.44**	±9.95
( *R*)-Pulegone oxide (**10**)	**43.21**	±2.31	**17.62**	±10.45	**16.02**	±6.43
(−)- *trans*-Isopulegone (**11**)	**18.96**	±5.08	**5.98**	±0.89	**7.56**	±6.73
(+)-Limonene 1,2-epoxide (**12**)	**73.13**	±2.77	**93.1**	±0.10	**58.48**	±1.07
(−)-Sobrerol (**13**)	**9.78**	±7.24	**41.4**	±4.20	**34.21**	±3.57
( *S*)-(−)-Perillyl alcohol (**14**)	**95.82**	±0.30	**91.68**	±7.06	**90.92**	±0.39
(−)-Perillaldehyde (**15**)	**83.03**	±1.54	**70.24**	±1.43	**59.28**	±5.78
(−)-Perillaldehyde 8,9-epoxide (**16**)	**98.64**	±0.74	**96.32**	±1.51	**99.89**	±0.24
(−)-Perillyl acetate (**17**)	**14.25**	±5.38	**3.06**	±2.34	**16.41**	±4.32
( *S*)-Perillyl benzoate (**18**)	**5.02**	±2.67	**2.86**	±1.72	**4.53**	±2.13
Doxorubicin	**99.24**	±0.15	**100**	±0.63	**99.57**	±0.31

Cell lines: OVCAR-8 (ovarian adenocarcinoma), HCT-116 (colon carcinoma), and SF-295 (glioblastoma) humans. GI% values are presented as the mean ± SD of three independent experiments measured by the MTT assay after 72 h of incubation. All compounds were tested at a concentration of 25 μg/mL. Doxorubicin was used as the positive control.

Evaluating the IC_50_ of compound **16**, besides presenting the highest percentage of inhibition of cell proliferation, **16** was more potent than **3**, showing high cytotoxicity in all three cell lines, with IC_50_ values ranging from 1.03–1.75 µg/mL, as shown in Table 2.

### 2.2. Hemolytic Assay

Since compound **16** showed high cytotoxicity in tumor cells, it was tested for its ability to induce lysis in mouse erythrocytes (data not shown). The mechanical stability of the erythrocyte membrane is a good indicator of *in vitro* damage in cytotoxicity assays, since many drugs can alter this delicate structure [37]. Red blood cells provide a preliminary model to study protective effects, substance toxicity, (or conditions associated with oxidative stress), where they are a possible indicator of such damage [38,39,40,41]. However, compound **16** was not hemolytic even at the highest tested concentration (500 μg/mL). This suggests that the cytotoxicity mechanism is probably related to a more specific pathway.

**Table 2:** Cytotoxic activity of (−)-perillaldehyde 8,9-epoxide and (−)-8-hydroxycarvotanacetone on tumor cell lines. Experiments were performed in triplicate.

Cells	Doxorubicin µg/mL	(−)-Perillaldehyde 8,9-epoxide µg/mL	(−)-8-Hydroxycarvotanacetone µg/mL
**HCT-116**	0.01 0.01–0.02	1.03 0.79–1.34	1.08 0.71–1.42
**OVCAR-8**	1.20 0.90–1.60	1.15 0.93–1.44	1.44 0.93–2.23
**SF-295**	0.24 0.17–0.36	1.75 1.05–2.93	3.24 2.47–4.26
**HL-60**	0.02 0.01–0.02	0.64 0.07–0.09	____

Cell lines: OVCAR-8 (ovarian adenocarcinoma), HCT-116 (colon carcinoma), SF-295 (glioblastoma), and HL-60 (leukemia) humans. Data are presented as IC_50_ values (μg/mL) and their 95% confidence interval obtained by non-linear regression from three independent experiments performed in triplicate, measured by the MTT assay after 72 h of incubation. Doxorubicin was used as the positive control.

### 2.3. (−)-Perillaldehyde 8,9-epoxide Inhibits the Proliferation of Human Leukemia in HL-60 Cells

**Figure 2:**
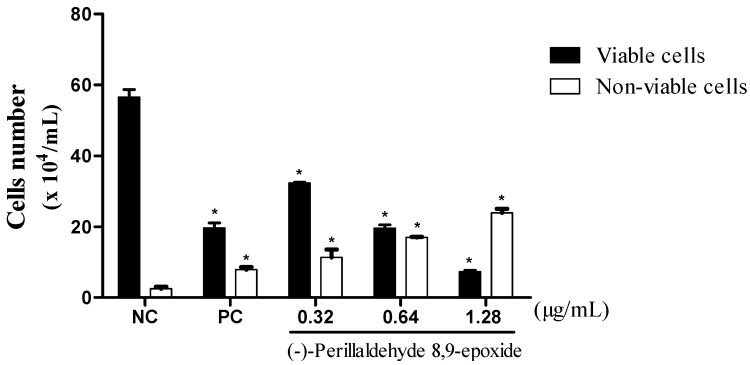
Effect of **16** on leukemic cell (HL-60) proliferation as measured by the trypan blue dye exclusion method after 24 h of incubation. The negative control was treated with the same vehicle (NC, 0.1% DMSO) used for diluting the test substance. Doxorubicin (PC, 0.3 µg/mL) was used as the positive control. Data are presented as mean values ± S.E.M. of two or three independent experiments performed in duplicate. * *p* < 0.05 compared to negative control by ANOVA followed by Student-Newman-Keuls test.

Three concentrations of compound **16**, ½ IC_50_ (0.32 µg/mL), IC_50_ (0.64 µg/mL) and 2 × IC_50_ (1.28 µg/mL) were chosen against HL-60. The anti-proliferative effects of **16** were demonstrated through the trypan blue dye exclusion test (Figure 2). It showed a reduction in the number of viable cells, and increases in the number of non-viable cells in a concentration-dependent manner after 24 h of incubation. The quantitative decrease in viable cells is common in cytotoxic compounds, as demonstrated by de Barros and collaborators [42], with a thiazacridine derivative, which decreases the number of viable HCT-8 cells in a concentration and time dependent manner.

### 2.4. (−)-Perillaldehyde 8,9-epoxide Induces Apoptosis and Necrosis in Human Leukemia HL-60 Cells

**Figure 3:**
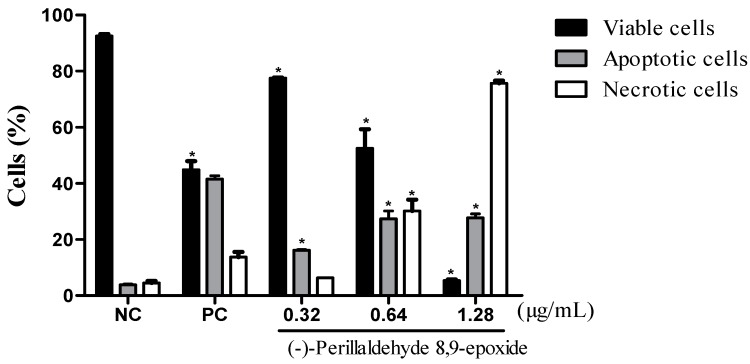
The effect of **16** on the viability of human leukemic cells (HL-60). Cell viability (viable cells-black bar; apoptotic cells-gray bar; and necrotic cells-white bar) was determined respectively by fluorescence microscopy using acridine orange/ethidium bromide after 24-h incubations. The data are presented as the mean values ± S.E.M. of three independent experiments performed in duplicate. The negative control was treated with the same vehicle (NC, 0.1% DMSO) that diluted the test substance. Doxorubicin (PC, 0.3 μg/mL) served as the positive control. * *p* < 0.05 compared to negative control by ANOVA, followed by a Student Newman-Keuls test.

Attempting to ascertain the cellular death process (as induced by **16)**, in cancer cells, two tests were performed, fluorescence microscopy using acridine orange/ethidium, and hematoxylin-eosin coloration analyzed by optical microscopy. After 24-h incubation, the effects of **16** were evaluated based on cell morphology using hematoxylin-eosin. All concentrations and showed severe drug-mediated changes. The hematoxylin-eosin stained HL-60 cells treated with **16** presented a morphology consistent with apoptosis, including a reduction in cell volume, chromatin condensation, and nuclei fragmentation, as well as, necrosis with membrane disruption, cell swelling and rupture leading to inflammation. The acridine orange/ethidium bromide stained and treated cells also displayed a morphology consistent with apoptosis and necrosis, in a concentration-dependent manner (*p* < 0.05) (Figure 3).

**Figure 4:**
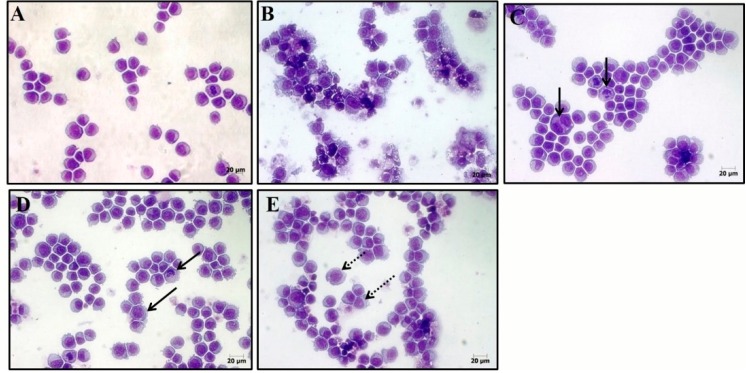
Effect of **16** on cell morphology for HL-60 human leukemia cells. The cells were stained with hematoxylin-eosin and analyzed by optical microscopy after 24 h incubation at concentrations of 0.32 (**C**); 0.64 (**D**); and 1.28 μg/mL (**E**). Negative control (**A**) was treated with the vehicle (0.1% DMSO) used for diluting the test substance. Doxorubicin (0.3 μg/mL) was used as the positive control (**B**). Continuous arrows show nuclear fragmentation and non-continuous arrows show accumulation of metaphases cells.

Compound **16** caused cell death by apoptosis in the three concentrations tested, as shown in Figure 3 and Figure 4. The induction of apoptosis is an important target in cancer therapy, achieved by compounds such as the vinca alkaloids, taxanes and colcichine [43]. It is the most well-known mechanism of cell death which functions to maintain homeostasis of the cells [44]. It is characterized by phosphatidylserine exposure, loss of mitochondrial membrane potential, caspase activation, chromatin condensation, nuclear fragmentation, and results in phagocytosis of membrane bound apoptotic bodies [45].

Besides the apoptotic process in doses of 0.64 and 1.28, compound **16** presented necrotic cell death simultaneously. This type of cell death is characterized by swelling of cellular organelles, cell membrane rupture, cell lysis (unlike apoptosis), and the core becomes distended yet substantially intact. Necrotic death is typically followed by inflammatory reactions [46]. This appears to be a limiting factor as the concentration of **16** is increased.

Various chemical agents such as β-lapachone described by Li and collaborators [47], induce cellular death by necrosis concomitant with apoptosis in a variety of cancer cells such as ovarian, colon, lung, prostate and breast cancer. Furthermore, some forms of treatment such as alkylating agents of DNA and photodynamic treatment are also able to induce cell death, activating necrosis [48]. Thus we might consider **16** as a possible candidate for *in vivo* tests, even with the two processes of cell death responsible for its *in vitro* cytotoxicity.

### 2.5. Structure-Activity Relationships (SAR)

This evaluation is important due to the fact that perillyl alcohol and its analogues are structurally similar to many of the chemical constituents of essential oils and other natural products. Therefore the results obtained in this study may well provide a reference for the development of novel cytotoxic agents having an appropriate biological profile.

The presence of an exocyclic aldehyde carbonyl and the conjugated C-C double bond seem to contribute to cytotoxicity, as (−)-perillaldehyde (**15**) displayed good cytotoxic activity, with a GI value of 59.28%–83.03%. Compounds **14** (absence of an epoxide group and replacement of an aldehyde by a hydroxyl group), and **15** (absence of an epoxide group and presence of an aldehyde function) were less cytotoxic (GI = 90.92%–95.82% and 59.28%–83.03%, respectively) than **16** (presence of both an epoxide and aldehyde group, GI = 96.32%–99.89%). This indicates that the presence of an epoxide and/or aldehyde function in the molecule is important for cytotoxicity. Further, the anti-proliferative activity was even more pronounced when these functional groups (epoxide and aldehyde) are both in the chemical structure, since (+)-limonene 1,2-epoxide (**12**) (GI = 58.48%–93.10%), which contains only the epoxide group, was less bioactive than **16** (GI = 96.32%–99.89%). On the other hand, comparing (+)-limonene 1,2-epoxide (**12**) with (−)-carvone epoxide (**5**), it appears that the endocyclic ketone function does not contribute to cytotoxicity, as compound **12** (GI = 58.48%–93.10%) was approximately 2.5- to 11-fold more cytotoxic than **5** (GI = 8.21%–29.24%).

The monoterpenes perillyl alcohol (**14**) and (−)-perillaldehyde (**15**) differ from each other only by a functional group. Compound **14** has a hydroxyl group whereas **15** presents an aldehyde function, both located at the same position in the *p*-menthane skeleton (C-7). Perillyl alcohol (**14**) (GI = 90.92%–95.82%) exhibited higher cytotoxicity compared to (−)-perillaldehyde (**15**) (GI = 59.28%–83.03%), suggesting that a hydroxyl group at C-7 position produces in a more potent inhibition of cell proliferation than an aldehyde group at the same position.

To investigate the effect of the disposition of the ketone carbonyl group in cytotoxic activity, we compared the oxygenated monoterpenes (−)-carvone (**1**) (GI = 2.28%–12.28%), (+)-carvone (**2**) (GI = 34.39%–48.07%), (+)-pulegone (**9**) (GI = 10.25%–27.44%), and (−)-*trans*-isopulegone (**11**) (GI = 5.98%–18.96%). In the general, the evaluated ketones showed moderate to weak cytotoxicity, but considerable differences in their potencies were observed. We also noted that compounds **9** (α,β-unsaturated ketone) and **11** (saturated ketone) displayed different cytotoxic effects against the three tumor cell lines.

Replacement of a carbon-carbon double bond, conjugating the carbonyl with an epoxide group resulted in a modest enhancement of anti-proliferative effect. Improvement of the biological potency was observed thru comparison of (−)-carvone (**1**) (GI = 2.28%–12.28%) with (−)-carvone epoxide (**5**) (GI = 8.21%–29.24%) and (+)-pulegone (**9**) (GI = 10.25%–27.44%) with (*R*)-pulegone oxide (**10**) (GI = 16.02%–43.21%). Similar results were found comparing the compounds (+)-carveol epoxide (**6**) and (−)-*cis*-carveol (**7**), and (−)-perillaldehyde (**15**) and (−)-perillaldehyde 8,9-epoxide (**16**). Replacement of a non-conjugated C-C double bond by an epoxide function in **7** (GI = 3.61%–21.16%) and **15** (GI = 59.28%–83.03%), resulting in the respective compounds **6** (GI = 4.58%–35.35%) and **16** (GI = 96.32%–99.89%), produces a subtle increase in cytotoxicity.

To ascertain the influence of the disposition of the epoxide and ketone groups in cytotoxicity, we compared (−)-carvone epoxide (**5**) with (*R*)-pulegone oxide (**10**). Compounds **5** and **10** present a ketone and epoxide group in different positions in the *p*-menthane skeleton, and different biological effects for these monoterpenes was observed. (*R*)-Pulegone oxide (**10**) was more bioactive (GI = 16.02%–43.21%) than (−)-carvone epoxide (**5**) (GI = 8.21%–29.24%), suggesting that the position of these groups in the *p*-menthanic structure influence the cytotoxic activity.

To examine whether the introduction of a hydroxyl group (hydroxylation of a double bond) in the molecular structure affects the cytotoxic activity, (−)-carvone (**1**) was compared with (−)-8-hydroxycarvotanacetone (**3**), and (+)-carvone (**2**) with (+)-8-hydroxycarvotanacetone (**4**). Compound **1** (GI = 2.28%–12.28%) was significantly less cytotoxic than **3** (GI = 61.59% to 94.01%), indicating that the presence of a hydroxyl group causes an enhancement of cytotoxicity. However, (+)-carvone (**2**) (GI = 34.39%–48.07%) was more bioactive than its corresponding monoterpene alcohol (+)-8-hydroxycarvotanacetone (**4**) (GI = 3.12%–16.36%). In addition, we also evaluated the effect of adding a second hydroxyl group to the *p*-menthane structure. It was observed that (−)-sobrerol (**13**) (two hydroxyl groups) showed a significant decrease in the inhibition rate of cell proliferation as compared to (−)-8-hydroxycarvotanacetone (**3**) (one hydroxyl group), with GI values of 9.78%–41.10% and 61.59%–94.01%, respectively. Thus, these results revealed that the more polar monoterpenes, such as (−)-sobrerol (**13**), are less effective inhibitors of cellular proliferation.

To investigate whether the presence of an ester function in the chemical structure alters the cytotoxicity, we compared (−)-8-hydroxycarvotanacetone (**3**) with (−)-acetoxycarvotanacetone (**8**), and (*S*)-(−)-perillyl alcohol (**14**) with both (−)-perillyl acetate (**17**), and (*S*)-perillyl benzoate (**18**). (−)-8-hydroxycarvotanacetone (**3**) (GI = 61.59%–94.01%) presented more potent cytotoxic activity than its acetate derivative, (−)-8-acetoxycarvotanacetone (**8**) (GI = 1.62%–30.47%), suggesting that the replacement of a hydroxyl group by an acetate causes a marked decrease in the cytotoxicity. Additionally, compound **14** was more cytotoxic (GI = 90.92%–95.82%) than **17** (GI = 3.06%–16.41%) and **18** (GI = 2.86%–5.02%), probably due to its lower lipophilicity. (*S*)-Perillyl benzoate (**18**), the most lipophilic compound, was found to be the least potent inhibitor of cellular proliferation for all of the cell lines evaluated.

To evaluate the influence of chirality on cytotoxicity, we compared the pairs of enantiomers (−)-carvone (**1**) and (+)-carvone (**2**), and (−)-8-hydroxycarvotanacetone (**3**) and (+)-8-hydroxy- carvotanacetone (**4**). (+)-Carvone (**2**) (GI = 34.39%–48.07%) was more cytotoxic than its enantiomer (−)-carvone (**1**) (GI = 2.28%–12.28%) in all the cancer lines tested. Similarly, (−)-8-hydroxy- carvotanacetone (**3**) exhibited better cytotoxic effect than its enantiomeric form (+)-8-hydroxy- carvotanacetone (**4**), with respective GI values of 61.59%–94.01% and 3.12%–13.36%. The findings suggest an association between the cytotoxicity of the monoterpenes tested and their stereochemistry.

## 3. Experimental Section

### 3.1. Chemical Analogues

The compounds (*R*)-pulegone oxide [49], (−)-carvone epoxide [50], (−)-*cis*-carveol [51], (+)-carveol epoxide [52], (+)-limonene 1,2-epoxide [53], (−)-perillyl acetate [54], (−)-perillaldehyde [55], (−)-perillaldehyde 8,9-epoxide [56], (−)-*trans-*isopulegone [57], (+)- and (−)-8-hydroxy- carvotanacetone [58], (−)-8-acetoxycarvotanacetone [59], (*S*)-perillyl benzoate [59], and (−)-sobrerol [60] were prepared in our laboratory according to the literature and analyzed by infrared, ^1^H- and ^13^C-NMR. (+)-Pulegone, (−)- and (+)-carvone, and (*S*)-(−)-perillyl alcohol were purchased from Sigma-Aldrich Co. (St. Louis, MO, USA). The chemical structures of the evaluated compounds are shown in Figure 1.

### 3.2. Cell Lines

The cytotoxicity of the 18 compounds was tested against OVCAR-8 (ovarian adenocarcinoma), HCT-116 (colon carcinoma), SF-295 (glioblastoma), and HL-60 (leukemia) human cancer cell lines, all obtained from the National Cancer Institute, Bethesda, MD, USA. Cells were grown in RPMI-1640 medium supplemented with 10% fetal bovine serum, 2 mM glutamine, 100 µg/mL streptomycin, and 100 U/mL penicillin, and incubated at 37 °C in a 5% CO_2_ atmosphere.

### 3.3. Cytotoxicity Assay

Tumor cell growth was determined by the ability of living cells to reduce the yellow dye 3-(4,5-dimethyl-2-thiazolyl)-2,5-diphenyl-2*H*-tetrazolium bromide (MTT; Sigma Chemical Co., St. Louis, MO, USA) to a purple formazan product, as described by Mossman [61]. For all experiments, cells were seeded in 96-well plates (0.1 × 10^6^ cells/mL in 100 μL medium). After 24 h, the 18 compounds (25 μg/mL), were dissolved in 0.7% dimethyl sulfoxide (DMSO), and were added to each well (three independent experiments, performed in triplicate). Then, the cells were incubated for 72 h at 37 °C in a 5% CO_2_ atmosphere. The experiment was performed as three independent experiments, using DMSO at 1%, and doxorubicin at 100 μg/mL as negative and positive controls, respectively. Doxorubicin, purity >98%; Sigma Chemical Co.).

At the end of incubation, the plates were centrifuged, and the medium was replaced by fresh medium (150 μL) containing 0.5 mg/mL MTT. Three hours later, the formazan product was dissolved in 150 μL DMSO, and absorbance was measured using a multiplate reader (DTX 880 Multimode Detector, Beckman Coulter Inc., Packard, ON, Canada). The treatment effects were expressed as the percentage of control absorbance of reduced dye at 595 nm. All absorbance values were converted into a cell growth inhibition percentage (GI%) by the following formula:

[GI% = 100 − [(T/C) × 100%]
(1)

where C is the absorbance for the negative control, and T is the absorbance in the presence of the tested compound.

The compounds that caused greater degree of cell growth inhibition and which have not been described in the literature for cytotoxic activity against tumor cell lines were evaluated for their median inhibitory concentration able to induce 50% of maximal effect (IC_50_). The same protocol for the same cells was used to determine the IC_50_, varying only the concentration of the compound tested from 0 to 25 μg/mL, to verify the most potent compound. In addition, the IC_50_ was determined against HL-60 only for the most cytotoxic compound **16**. Compounds with an IC_50_ value lower than 4 μg/mL were considered promising for the search for new anticancer agents [62].

### 3.4. Hemolytic Assay

The test was performed in 96-well plates using a 2% mouse erythrocyte suspension in 0.85% NaCl containing 10 mm CaCl_2_, following the method as described by Jimenez and collaborators [63]. Various concentrations of compound **16** (0–500 µg/mL) were added to the suspension of red blood cells obtained from mice according to methodology adapted from Kang and collaborators [64], Pita and collaborators [65], and Bezerra and collaborators [66]. The tubes with the compound **16** erythrocyte mixtures were incubated on a mixer for 60 min and then centrifuged at 3000 rpm for 5 min. Mixtures were incubated on a mixer for 60 min and then centrifuged at 3000 rpm for 5 min. After incubation at room temperature for 30 min and centrifugation, the supernatant was removed and the liberated hemoglobin was measured spectrophotometrically as the absorbance at 540 nm.

### 3.5. Cell viability—Trypan Blue Dye Exclusion Test

The cell viability was determined by the trypan blue dye exclusion test. HL-60 cells were seeded (0.3 × 10^6^ cells/mL) in absence or presence of different concentrations of compound **16** (0.32, 0.64, and 1.28 µg/mL). After the incubation period of 24 h, 90 µL were removed from the cell suspension and added to 10 µL of trypan blue. Viable and non-viable cells were differentiated and counted in a Neubauer chamber [67].

### 3.6. Morphological Analyses Using a Fluorescence Microscope

HL-60 cells were pelleted and re-suspended in 20 µL of PBS. Then, 1 µL of aqueous acridine orange/ethidium bromide solution (AO/EB, 100 µg/mL) was added, and the cells were observed under a fluorescence microscope. Three hundred cells were counted per sample and classified as viable, apoptotic or necrotic [68].

### 3.7. Morphological Analysis with Hematoxylin-Eosin Staining

For morphological cell analysis, we used hematoxylin-eosin and examined using light microscopy. HL-60 cells were seeded (0.3 × 10^6^ cells/mL) in the absence or presence of different concentrations of compound **16** (0.32, 0.64 and 1.28 µg/mL). After an incubation period of 24 h, 50 µL of cell suspension were transferred to cytospin slides, fixed with metanol for 60 s, and stained with hematoxylin-eosin [43].

### 3.8. Statistical Analysis

Data are presented as mean ± SEM (or SD) or IC_50_ values, and their 95% confidence intervals (CI 95%) were obtained by nonlinear regression. Differences among the experimental groups were compared by one-way variance analysis (ANOVA), followed by Newman-Keuls test (*p* < 0.05). All analyses were carried out using the Graphpad program (Intuitive Software for Science, San Diego, CA, USA).

## 4. Conclusions

Based on the data of this study, we can conclude that among the 18 derivative compounds of perillyl alcohol tested, compound **16** was the *p*-menthane derivative with the highest cytotoxic activity against the cancer cells lines tested. The process induced apoptotic and necrotic cell death. In addition the analysis of structure-activity relationships demonstrated that the greater anti-proliferative activity of **16** was determined by chemical aspects, such as presence of functional groups and their positions on *p*-menthane skeleton, suggesting that using appropriate structural modifications in these compounds, it may be possible to develop new cytotoxic agents. Therefore, compound **16** is a potential anticancer drug, yet requiring further tests to determine its *in vivo* antitumor activity.
